# R-group replacement database for medicinal chemistry

**DOI:** 10.2144/fsoa-2021-0062

**Published:** 2021-06-30

**Authors:** Kosuke Takeuchi, Ryo Kunimoto, Jürgen Bajorath

**Affiliations:** 1 Department of Life Science Informatics, B-IT, LIMES Program Unit Chemical Biology & Medicinal Chemistry, Rheinische Friedrich-Wilhelms-Universität, Friedrich-Hirzebruch-Allee 6, Bonn, D-53113, Germany

**Keywords:** analogue series, compound optimization, data repository, network data structure, replacements, R-groups

## Abstract

**Aim:**

Generation of an R-group replacement system for compound optimization in medicinal chemistry.

**Materials & methods:**

From bioactive compounds, analogue series (ASs) were systematically extracted and from these ASs, all R-groups were isolated and further analyzed.

**Exemplary results & data:**

From more than 17,000 ASs, more than 50,000 unique R-groups were isolated. For the 500 most frequently used R-groups, preferred replacements were identified and organized in hierarchies. All original data and an R-group replacement database are made available in an open access deposition.

**Limitations & next steps:**

The searchable database has no limitations and can easily be modified using the source data we provide. The next step will be applying this R-group resource in practical medicinal chemistry projects as decision support.

## Lay abstract

To optimize the biological activity of small molecules in medicinal chemistry, series of analogues are generated by introducing substituents (R-groups) at different positions. The choice of R-groups largely depends on the experience of individual chemists. We have computationally isolated a large number of R-groups from currently available analogue series. Frequently used R-groups and their preferred replacements were identified and organized in a searchable database for medicinal chemists to aid in R-group selection.

## Graphical abstract

For a representative frequently used R-group (blue, left), four pathways with a preferred first (green) and second (pink) replacement are depicted (from left to right). Such R-group replacements were systematically derived from thousands of series of bioactive compounds.

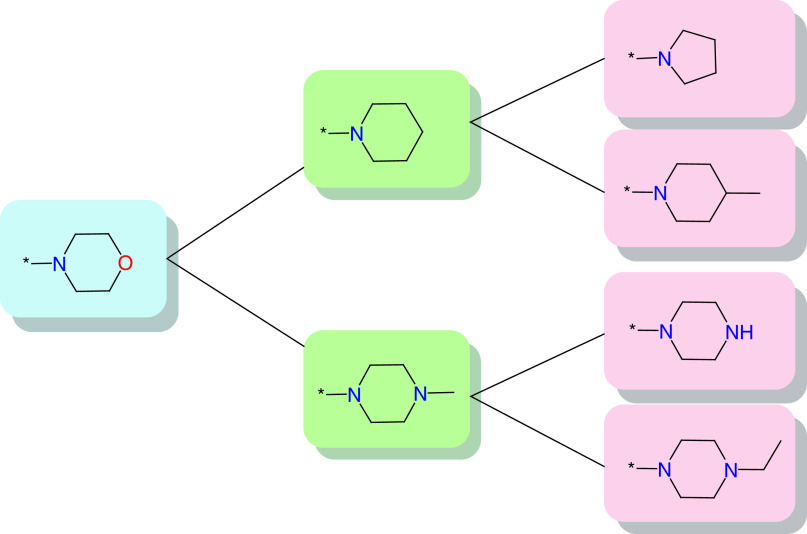

Compound optimization is of central relevance in the practice of medicinal chemistry. During hit-to-lead and lead optimization, analogue series (ASs) are generated from selected active compounds by iteratively modifying the core structure through the introduction of substituents (R-groups, functional groups) at different sites. These chemical optimization efforts aim to improve compound potency and other molecular properties such as solubility or *in vivo* characteristics. Core structures (scaffolds) of different compound classes have been extensively investigated from different perspectives [1–3], but the situation is different for R-groups. Here, the main focus has been on identifying bioisosteric replacements [4–6], which are supposed to retain biological activity of analogues but modulate other optimization-relevant properties in a favorable manner. Apart from the analysis of bioisosteres, only few studies have investigated R-groups in a general way. For example, algorithms have been introduced for extracting R-groups from individual compounds [7] or ASs [8] and two recent studies have systematically identified R-groups across currently available bioactive compounds [8,9]. We have further extended the assessment of R-groups on the basis of ASs [8] by generalizing the approach and identified all R-groups that currently occur in ASs (unpublished results). As a part of this study, we have generated a searchable database of frequent R-groups and their preferred replacements. To our knowledge, the database represents the first R-group replacement resource of its kind. Herein, we introduce this database, describe its derivation and report an open access deposition making it freely available for medicinal chemistry applications.

## Materials & methods

### Bioactive compounds & ASs

**Figure 1.**
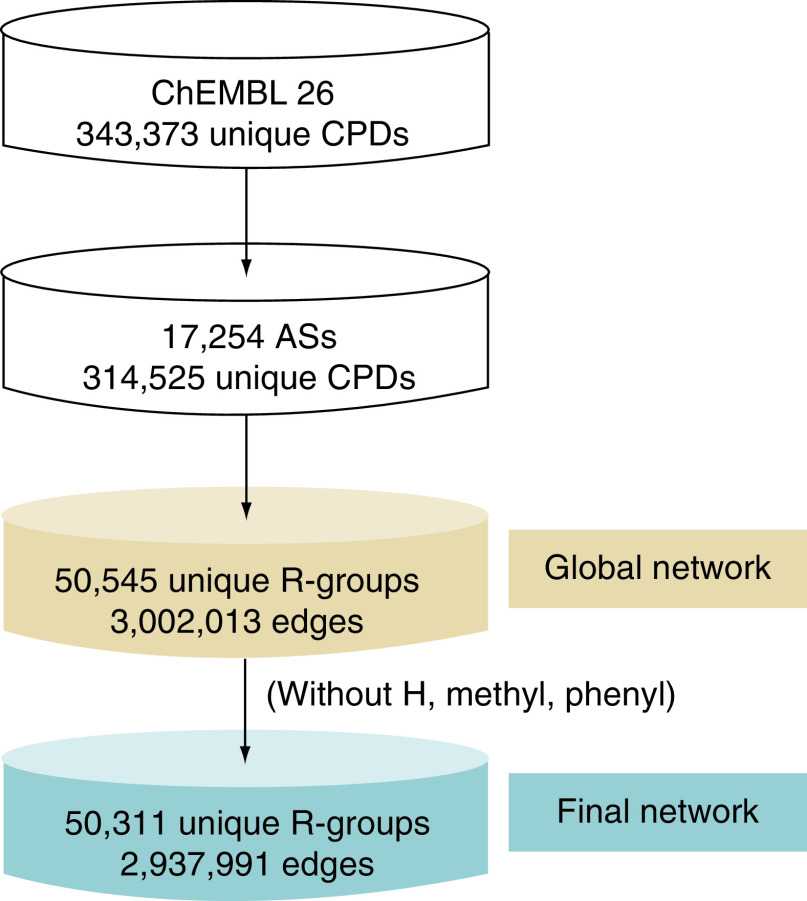
From analogue series to a comprehensive R-group network. Statistics are provided for the selection of bioactive compounds, identification of ASs and generation of the global and final R-group network. AS: Analogue series.

ASs provide a relevant structural context for R-group exploration. To comprehensively map R-group space of bioactive compounds at the level of ASs, compounds with different activity annotations were combined. Bioactive compounds with molecular weight ≤1000 Da were selected from ChEMBL (release 26) [10]. These compounds were required to be tested in direct binding/interaction assays (relationship type: ‘D’) against individual targets at the highest assay confidence level (assay confidence score: 9). Only numerically specified K_i_ or IC_50_ values were accepted as activity annotations (approximate values were disregarded). On the basis of these selection criteria, at total of 343,373 compounds were obtained and recorded as simplified molecular-input line-entry system (SMILES) representations [11]. Using this compound pool, ASs comprising at least three analogues were systematically identified using the compound–core relationship (CCR) method [12]. The CCR approach fragments compounds according to retrosynthetic criteria, leading to the identification of ASs with indexed substitution sites [12]. Importantly, to generalize the approach and identify all possible R-groups, beyond those defined by retrosynthetic rules, we introduced an algorithmic CCR variant in which retrosynthetic fragmentation was replaced by random fragmentation of single exocyclic bonds in compounds. Applying this algorithmic CCR variant, a total of 17,254 ASs were identified consisting of 314,525 unique compounds, with a median value of eight analogues per series (Figure 1). The core structure of an AS was required to contain at least half of the nonhydrogen atoms of each of the compounds forming the AS. The size of an R-group was limited to maximally 13 nonhydrogen atoms.

### Sampling of R-groups & replacements

From each of the 17,254 ASs, the core structure was isolated. Taken together, the core structures contained a total number of 61,312 indexed substitution sites. For each substitution site, all R-groups were sampled, leading to the identification of a total of 50,545 unique R-groups. For comparison, retrosynthetic CCR processing yielded 40,034 R-groups under the same fragment size restrictions. Hence, the generalized approach identified many more R-groups. For each substitution site, all possible R-group replacements were determined in a pairwise manner. For example, four R-groups found at a given site yielded six pairwise (bidirectional) replacements. In total, 3,002,013 unique R-group replacements were detected. The frequency of each replacement across all 61,312 substitution sites was determined. Importantly, the R-group replacement analysis was substitution site specific. Thus, each recorded replacement occurred at a given substitution site in an AS. This criterion ensured that R-group replacements were only considered if they took place at a given site in an AS, in other words, within the same chemical context. Then, all site-specific replacements were combined. Potency alterations as a consequence of individual AS-specific R-group replacements were not considered because these replacements were recorded across all ASs and their biological activities. The calculations were carried out with in-house generated code and the aid of RDKit [13] and the OpenEye Toolkit [14].

### R-group network analysis

The very large volume of R-group and replacement data was further analyzed using a network structure in which nodes represented individual R-groups and edges pairwise R-group replacements that were detected. Accordingly, the global network contained 50,545 nodes and 3,002,013 edges (Figure 1). For the global network and a further refined variant (described below), two network parameters were calculated including the node degree (ND) accounting for the number of edges per node and the edge weight (EW) reporting the frequency with which each unique replacement occurred over all substitution sites. Accordingly, ND and EW served as a measure of frequently used R-groups and replacement frequency, respectively. The R-group network data structure was generated and analyzed with the aid of Cytoscape [15].

## Exemplary results

### Frequently used R-groups

**Figure 2.**
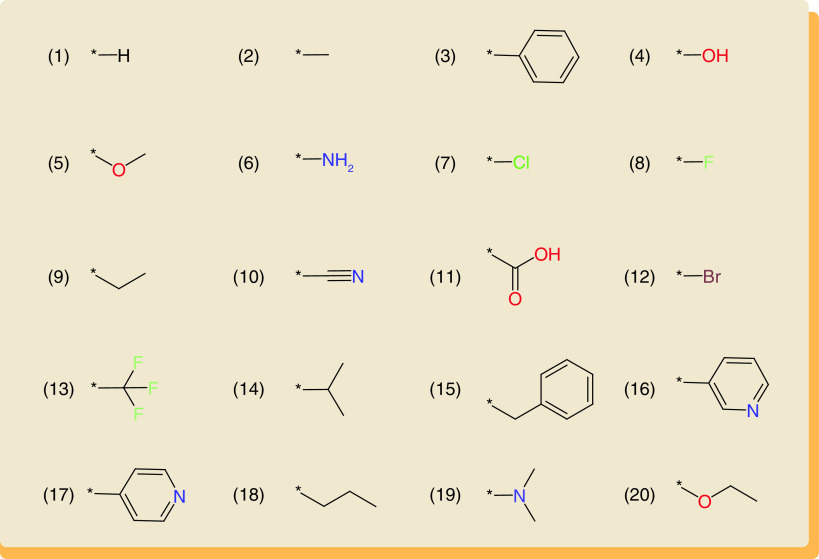
Frequently used R-groups. The top 20 most frequently used R-groups from the global network are shown in standard atom coloring. *Attachment points.

R-groups in the global network were ranked according to their ND values. Figure 2 shows the 20 most frequent R-groups. All of these R-groups are popular in medicinal chemistry, as expected. The overall most frequently detected hydrogen atom was a special case, because it was consistently present at each substitution site prior to a replacement and involved in a replacement when another R-group was introduced at this site. We analyzed the frequency with which individual R-groups occurred across all replacements in the global network and found that the top three R-groups in Figure 2 including the H atom, methyl group and phenyl ring were for the most part involved in replacements of other frequently used R-groups. Therefore, to avoid a domination of preferred R-group replacements by these three most frequent groups, we generated a further refined network variant in which the H atom, methyl group and phenyl ring were omitted, leading to the final R-group network (Figure 1). In the final network, which contained 50,311 nodes and 2,937,991 edges, the ordering of the most frequent R-groups remained constant (beginning with the hydroxyl group at position 4 in Figure 2).

### Preferred R-group replacements

**Figure 3.**
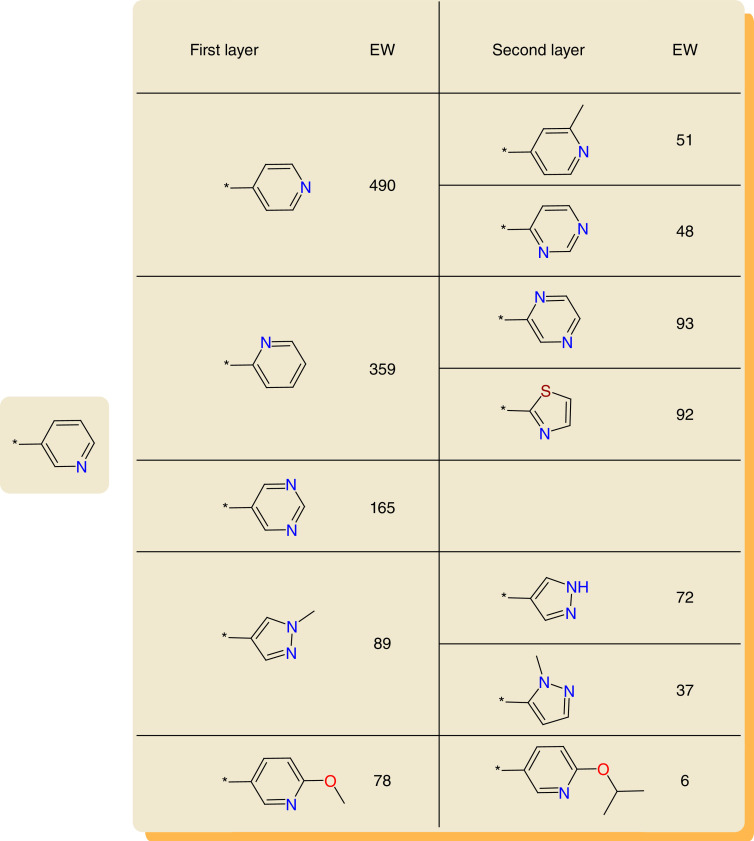
R-group replacement hierarchy. An exemplary 5 × 2 R-group replacement hierarchy is shown. For a frequently used R-group (left), preferred replacements (and their edge weight values) are reported. EW: Edge weight.

From the final network, preferred replacements of frequently used R-groups were derived on the basis of EW values. To this end, first and second layer replacements were considered. First layer replacements involved immediate network neighbors of a frequent R-group and second layer replacements nearest neighbors of first layer groups. Based on this layer concept, a variety of R-group replacement hierarchies can be generated. We focused our analysis on a 5 × 2 first and second layer replacement data structure. Accordingly, for each frequently used R-group, first and second layer replacements were ranked by EW values (accounting for the frequency of occurrence) and the top five first layer replacements were selected. For each of the first layer replacements, the top two second layer replacements were then recorded (if available). Figure 3 shows a representative example of this data structure, which defines a maximum of 10 first and second layer replacements sequences for each frequent R-group. We generated 5 × 2 replacement hierarchies for the top 500 most frequent R-groups. Of note, due to the removal of the top three R-groups from the global network, the methyl group and phenyl ring can be considered as additional generic replacements in all hierarchies (not taking into consideration the special H atom case).

### Local network view

**Figure 4.**
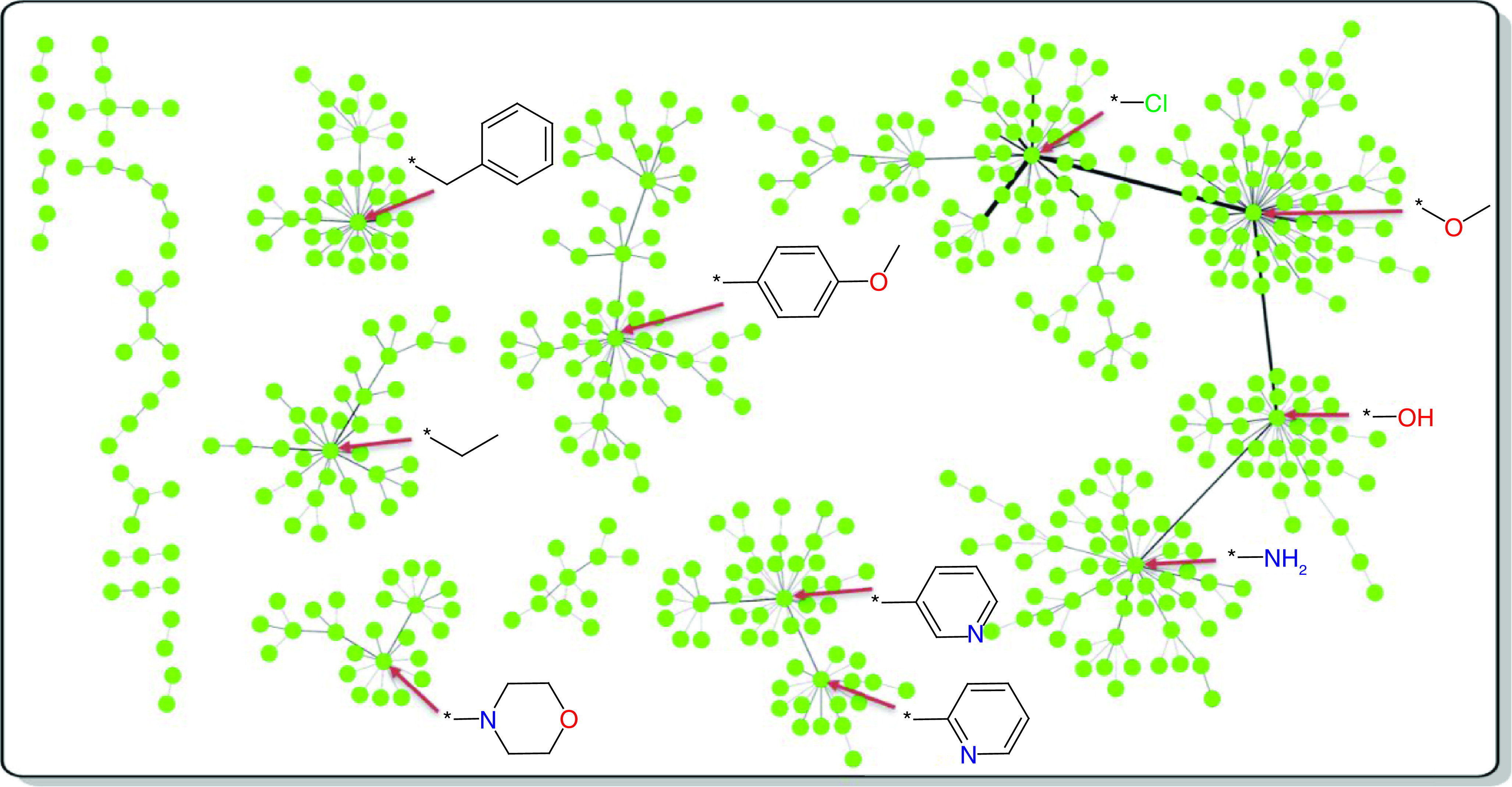
Local R-group network. Shown is a local network representation for the 500 most frequent R-groups (green nodes) extracted from the final network. The structures of the 10 R-groups with largest node degree values in the local network are depicted and assigned to their nodes (using red arrows).

While the global and final R-group network were too large and complex for display, we have also generated a local network variant only considering the 500 most frequently used R-groups (Figure 4). Compared with the global or final network, this small local network has a dramatically reduced edge density (and is used for representation purposes only). It provides a view of replacements among most frequent R-groups. In the local network, central ‘hubs’ emerge (Figure 4) representing R-groups with largest ND values in this network.

## Data

We provide a searchable repository containing the 500 most frequently used R-groups from the final network and their 5 × 2 first and second layer replacement hierarchies. In addition, the final network data are made available such that other R-group replacement hierarchies can also be generated.

### Network data structure

The 50,311 unique R-groups and 2,937,991 pairwise replacements comprising the final network are provided as a comma-separated values (CSV) file containing three columns (source, target and weight) specifying the weighted edges. Given the large number of edges, filtering by EW is advised prior to using the data as input for Cytoscape [15].

### R-group replacement database

The 500 most frequently used R-groups from the final network and their 5 × 2 replacement hierarchies are provided in a searchable text file in EXtensible Markup Language (XML) format [16]. R-groups are recorded as SMILES strings. Using this database, for an R-group of interest, preferred replacements across bioactive compounds can be identified by searching for the R-group and viewing the corresponding replacement hierarchy. The data deposition contains an example.

### Data deposition

The data have been deposition on the ZENODO open access platform [17]. The deposition also includes a readme.txt file with data file descriptions and an R-group search example using XML.

## Limitations & next steps

The R-group replacement database has no intrinsic limitations. It is based on a comprehensive assessment of R-groups and can easily be accessed and modified by a user. We also make all original R-group network data available in the open access deposition from which frequently used R-groups and their preferred replacements were derived. This makes it possible to generate additional or other replacement hierarchies. Of note, because we aimed to cover global R-group space as comprehensively as possible, the underlying molecular fragmentation approach was generalized and not restricted by predefined reaction information; hence, it yielded a very large number of R-groups. Consequently, corresponding chemical reagents might not be available for any formally defined (but rarely used) R-group or might be limited in a number of instances. However, the network data structure contains all of the commonly used R-groups in medicinal chemistry and many more and thus represents a rich knowledgebase. The next step will be applying the database in practical compound optimization to help select R-group replacements in an iterative manner. Since the underlying R-group analysis was substitution site centric and covered a very large number of substitution sites across bioactive compounds, there should be a meaningful balance between utility and novelty of R-group replacements. We hope that the new R-group resource will be of interest and use in medicinal chemistry.

**Summary points**

**Background**

- Compound optimization is discussed.
- Bioisosteres and systematic R-group analysis are introduced.

**Methodology**

- Algorithmic generation of analogue series is described.
- Identification of frequent R-groups and preferred replacements is detailed.
- R-group network analysis is introduced.

**Exemplary results**

- Frequently used R-groups are identified.
- R-group replacement hierarchies are generated.
- A local R-group network view is provided.

**Data**

- The network data file and R-group replacement database are described.
- The open access data deposition is detailed.

**Limitations & next steps**

- For rarely used R-groups, reagents might be limited.
- Practical applications of the R-group resource are encouraged.
